# Cases of Syphilis

**Published:** 1870-02

**Authors:** F. B. Norcom

**Affiliations:** Chicago


ARTICLE IV.—*Cases of Syphilis.* By F. B. NORCOM, A.M., M.D., Chicago.

CASE No. 1.—Mr. L——, unmarried, æt. 29, came to consult me, June 3, 1868, in relation to ulcers near the frænum. Muscular; full of red blood; nervo-bilious temperament; fair in his habits; moderately addicted to wine, a little more so to women. He had contracted his present trouble from an intercourse dating fifteen days back, about 19th May, and it was now the ninth day of its appearance. An examination revealed two small chancroids, right side; the smaller invading the frænum, and still spreading at its periphery; the larger showing some slight reparative action; both irritable, and covered by a rather profuse purulent discharge. No specific induration could be detected, and both groins exhibited multiple enlargements, the right, however, with large single bubo, tender upon pressure, and evincing a desire to suppurate. There had appeared, also, a slight watery discharge, a few days before, from the urethral canal, with some pain, and being already alarmed at the delay in his cure, this new complication caused him to dismiss unwarrantably his surgeon, and apply to myself, a change I did not believe for the better. I could discover nothing in the canal except a hypersensitiveness upon passing the sound, and simply recommended mild astringent injections. His general condition was not good; tongue coated; bowels costive; anorexia; light fever, with decided exacerbation at night, and broken slumber. Though very anxious about himself, he was unwilling to incur much further expense, nor could be persuaded to leave his business and keep quiet until the ulcers were healed, and all tendency to glandular suppuration averted. I touched the periphery of each chancroid with nitric acid, and ordered an astringent wash with aromatic wine, at same time attending to his proper secretions, and giving him a quinine and acid tonic. I explained the folly of keeping so constantly on his feet, and undergoing the fatigue of business, if he desired a speedy and happy termination to his present trouble.

Did not see him again until June 16, when I visited him at his boarding-house, to find that he had been under the management of one of his personal friends, who had had some experience of his own; had read Ricord, and who, though not a medical man, considered himself capable. To each groin a large, soft, fluctuating tumor, the right one about to open spontaneously; frænum destroyed; chancroids partially cicatrized, though indolent; and general condition bad, there being great constitutional irritability, with its usual concomitants. Gave him an anæsthetic; opened both buboes freely, and with my thumb gouged out the broken-down gland structure, making as clean cavities as practicable, and using water dressing covered with oil-silk, at same time slightly brushing the chancroids with nitrate of silver.

The next day, the 17th, he felt refreshed, a good night's rest being secured by an anodyne, and having lost that dreadful, dull, throbbing pain and fullness of the groins. Under a liberal and nutritious dietary, with tonics, he began to build up rapidly, and I left the case, 12th July — the cavities having almost closed — assuring him that there was no constitutional infection.

On 5th September he reappeared, with syphilitic roseola of the forehead; faucial erythema; some degree of syphilitic fever; loss of appetite; and in a condition of extreme alarm and rage, partly directed towards his disease and its future bearings, but more particularly against his doctor, who should have foreseen all this, and provided against its approach. It was somewhat puzzling to account for these secondary manifestations, especially as he had been continent since his first attack, and I felt certain my diagnosis and treatment had been correct and philosophical. Both groins exhibited well-marked multiple enlargements; posterior mastoideon adenitis also present, but no nodulation at the site of the chancroids. I examined by palpation and with the instruments the canal critically, to discover if possible the situation of the chancre, which must have been a companion to the ulcers, but failed. Believing in the spontaneous elimination of syphilis under favorable circumstances, and knowing my patient to have a fine constitution, I calmed his fears, and ordered mercury and quinine to simply touch his gums. This was accomplished in about four days, then the mercury remitted, and tonic continued for at least a month, besides a nourishing diet, etc., etc. He called upon me several times this month, and everything seemed satisfactory, the eruption fading rapidly, and the congestion of the throat improved, having never reached abrasion.

On the 11th October I renewed the mercurial, always combined with tonics, liberal food, warm bathing, etc., etc., and having again touched the gums, dropped the drug, but continued the sustaining treatment, though at this stage there was present a lenticular eruption of the abdomen, a few impetiginous spots of the scalp, and some alopecia.

He left the city shortly after this, and I did not again see him until the first week in January, 1869, when he was suffering from mild iritis, of few days standing; sore throat, superficial ulceration; several mucous patches on the tongue; and osteo-cepic pains at night. The iodide potassium in compound tincture cinchona; mercurial inunctions, etc., were exhibited, while the photophobia was greatly relieved by daily painting the forehead with strong tincture iodine, and throat washed with 40 gr. solution nitrate silver. Under these remedial agents he recovered rapidly, and up to this date — for I have watched him carefully, and always kept him in sight — has had no further accidents, and enjoys very robust health.

I present this case merely as a type of a certain class coming under the observation of physicians, and appearing subversive of the generally received doctrine, that constitutional contamination can only follow chancre. From observation, and from a critical examination of such "mixed cases," I have, as a rule, been able to discover that the true syphilitic sore did exist; and in several instances where I procured examinations of the women, have generally determined, if not the ulcer itself, the evidence of systemic poisoning, as shown by the engorged glandular system, the cutaneous irritation, and the morbid changes of mucous surfaces.

That a concealed urethral chancre (*larvé*) did exist in this instance, I have no doubt, there being discharges and pain for several days, but escaped from my want of skill in detecting its *locale*.

The present and subsequent history of his *inamorata* I could never learn, as he was unwilling to renew the acquaintanceship; nor could he be persuaded to prosecute further inquiries as to her present condition, she being a "ladye faire," blushing under the shade and shadow of her husband's "vine and fig-tree," and doubtless ignorant that her *complacency* and *abandon* had given birth to such sad results among at least one of her admirers.

Moral: *Le jeu ne vout pos la choudelle.*

Case No. 2.—Mr. B., aged 26 years, unmarried, a book-keeper, nervo-bilious temperament, and with strumous tendencies from hereditary taint, formerly much given to social enjoyments — pretty free use of alcoholic stimulants; ditto of females — *comibus longissimis*, *et id omne genus* — and consequently had received several visitations from clap.

He was placed under my care May 2, 1868, suffering from two putative chancroids on prepuce, near and to right of frænum, also multiple adenitis; and as the treatment so far pursued had been judicious, and the ulcers granulating, it was thought all danger of glandular suppuration had passed, and there could be no constitutional poisoning. His general condition was good, and there appeared nothing to do but continue, and allow the sores to heal. I was convinced, however, the moment I pressed the ulcers between my fingers, and from their extremely superficial character, as well as almost entire absence of discharge or secretion, that I had not chancroids to deal with, but well-marked parchment chancres, and was still strengthened in this belief by the long period of incubation — thirteen or fourteen days — and by the attendant *pléaides ganglionaires.*

As the case was now placed fairly under my charge, I prescribed a mild stimulating lotion, and such measures as would tend to keep up the tone of his system; at same time administering iron and tonics to prevent, as far as possible, blood deterioration. I was forced to give my opinion that, within three months, he must expect constitutional manifestations, and caution him against excesses, especially the too free indulgence in liquors, etc., which might tend to impair his vital or recuperative power, as I should count largely upon this to assist me in eliminating the *materies morbi*, when the time came for active interference. In ten days his ulcers were healed, and all tenderness of the inguinal region subsided, and his treatment continued.

In July he returned, with inflammation of fauces and tonsils, mottled and rough; slight falling of the hair; a well marked papular eruption of both legs; and some constitutional disturbance.

His face had lost, in some degree, its fresh, rosy hue, and he had fallen in weight, showing the sure and subtle influence of this specific virus upon his general nutrition.

He was given the blue mass, quinine, and opium until its effects were noticed upon his gums, then discontinued; and tonics, besides a liberal dietary, with a glass or two of wine or ale throughout the day; bathing, and exercise in the open air, so far as compatible with his business. This management was continued steadily, the mercurial resorted to every four or six weeks, for a few days, for at least four trials; then a course of alkaline carbonates, with bitter tonics, to complete the course. The eruption had gradually disappeared, leaving the characteristic coppery or violacious stains — so pathognomonic of this disease — and all the attendant secondary changes ameliorated.

The October following, he came in from one of our western cities, suffering acutely, the last few days, with iritis of the left eye, sympathetic weakness and irritation of its companion, and severe frontal neuralgia. Dr. E. L. Holmes saw the patient with me, and as abundant lymph had been thrown out, covering the iris and occluding its pupil, he advised the treatment, already instituted, to be continued — mercurial inunctions, iodide potassium, quinine and atropia, and counter-irritation locally.

The inflammatory condition passed away in two weeks, leaving, however, great weakness, and some blurring of vision from defective nutritive changes in the atroptric media; but in a few months, these symptoms passed away; and under the continuous use of iron, quinine, cod-liver oil, etc., he seemed to regain his health; and by the spring, weighed as heavily as usual.

This treatment was essentially demanded by the condition of his lungs, which exhibited, upon careful examination, crude tuberculous masses in their apices; harsh inspiration; p·olonged expiratory murmur; increased vocal fremitus and resonance; with dry cough and shortness of breath. Even these symptoms diminished in intensity, and in June his physical condition was better than it had been for many years.

On 11th August, while walking in the street, he was suddenly struck by hemiplegia, being only preceded the night and morning before by some little vertigo, distaste for food, and a feeling of *malaise*. I saw him a few hours after his seizure; found complete palsy, hyperæsthesia, and, for a short time, increased temperature of the left side, with well-marked reflex movements; no abnormal intercranial sensations, but palsy also of same side of face, tongue, and enlarged pupil. Prof. Moses Gunn came to consult with me, and though we could not decide upon the exact lesion, yet, without question, we considered this state to result from specific disease.

Cold and heat applied alternately to the spine; large doses iodide potassium; moderate diet; absolute quietude; and, to satisfy every demand of his anxious friends, the direct current of electricity.

On the 19th, he had so far recovered that he left the city, to stay with some relatives; and since that time has been steadily improving, regaining his lost powers, and exhibiting no further manifestations of a syphilitic character. He is now in fine order, still taking his tonics, cod-liver oil, etc., and giving every attention to his general health. "We shall see what we shall see."

This patient presents to us several facts worthy of attention; the main one, the early invasion of the intercranial structures; the marked severity of the seizure; and the celerity of his recovery therefrom. As a rule, such attacks occur at a much later period of the disease, and come within the category of tertiary accidents, due either to slowly-formed periosteal growths, with pressure, or to *ramolissement* engendered by deficient red-blood supply. We have the analogue in these so-called atheromatous changes produced by gout, rheumatism, etc.

Notice the symptoms, in their proper sequence, between case number 1 and case number 2: Both young men, with plenty of recuperative energy; the one, however, with hereditary predisposition to scrofula, and impaired *vital power from excesses in alcohol*, women, and irregular hours; the other, a perfect constitution and well regulated life. In the latter, the secondary symptoms of the mildest character, easily controlled, and never bordering upon the tertiary stage; in the former severe secondary, rapidly assuming tertiary forms, affecting the most important organs of the economy. Elimination and antagonism rapid and well defined in the one; feeble and unpromising as to the future in the other.

Moral: Live temperately, and husband your resources like a Christian; be virtuous, and you will be happy.

Case 3.—Col. H., married, aged 51, of sanguine temperament, vigorous and full-blooded, came to consult me Aug. 20, 1868, for a swelling in his left groin, somewhat painful upon pressure, and so far impeding locomotion as to interfere with his daily business; of irregular habits, being fond of his *toddy*, equally so of high living, and still more so of those frail daughters, who "submit not without a sigh, and yet submit." Never been afflicted by any form of venereal disorder before.

The left groin was occupied by a very hard and tender tumor, and accompanied by numerous smaller satellites; the right, with several well defined enlargements, also; no throat trouble; no posterior cervical adenitis; nor could I detect the *debris* of an ulcer on the organ, nor elicit any information from a strict examination of the canal.

He only noticed the swelling about six or seven days before, and he assured me that he had held no intercourse with women since his wife's departure for the East, about the latter part of June. I was the more inclined to accept this statement, as he was an upright man, a member of the Board of Trade, went to church habitually, and was kind and considerate to all brought in contact with him; so, I had to pursue or fall back upon some of those far-fetched theories entertained by the profession many years, even after Fernel, when our knowledge of syphilis was in its infancy, and when it was supposed possible to contract it by mere contact — the shaking of hands, the chaste salute, etc., — bearing the seeds of contagion, like the glove, or the bouquet, or the ring of a Borgia. His general condition was not particularly bad. Some little constitutional irritation, engendered, or at least enhanced, by his daily potations, for he was pretty *full* at this moment. He was ordered saline cathartics; his diet lowered; confined to his sofa; cut off from his toddies as far as possible; painted with strong tincture iodine; and sponge compress, with spica bandage, applied. He was visited at his house for ten days, the iodine and compression decidedly persisted in, and the bubo soon diminished to almost the size of its companions, when he was placed upon iron, quinine and small doses of protiodide, and at same time resumed his business, and, in all probability, his ordinary habits. I believe he continued this treatment not longer than three weeks, and then thought himself cured; but September 29th, came back, with hoarseness and faucial inflammation, in patches and abrasions, extending to the tonsils; lenticular eruption of the belly, and syphilitic efflorescence of the forehead and right cheek. His system in a measure sympathized with these manifestations, and his mental condition was distressing. He was placed under strict constitutional treatment, and all proper means instituted to keep up a fair balance between his health and an attempted elimination or neutralization of the specific virus circulating in his blood. On the 4th of October, the proper effects being noticed back of his molars, the mercurial was much reduced in its dose, and a nutritious but unirritating regimen instituted, with small quantities of sound wine to replace strong drinks.

As the colonel felt very wretchedly about having such a disorder, the more especially as he could not understand how it had been contracted, and might lay him open to the animadversions of his friends, should he be suspected, he followed the treatment ordered rigidly and faithfully, confining himself to the house until at least his face could no longer be the tell-tale of his "unmeasured woes," and, by letter, retarded the return of his wife, expected home about the middle of the month. This step appeared to me rather unnecessary, as this gentleman had repeatedly assured me of his entire innocence, and should not have been apprehensive. Ah, colonel! should I accept your statement without the *granum salis*, it would knock all my long-cherished theories, all my hard-worked experience into a cocked hat, and throw a mazy veil of obscurity and doubt over the results of many years of untiring labor and accurate observation; but ponder and read:

> I saw the loveliest rose that graced the lawn,
> With blooming fragrance gladdening all around:
> Too bold, I thrust the forward hand,
> Missed the fair flower, and only felt the wound."

The eruption on his face, a delicate roseola, began to subside in a few days, but impetigo appeared on his scalp and posterior cervical enlargements; while his throat became, if anything, worse, the ulcers deeper, more confluent, and more characteristic, and voice almost entirely gone. A rapid and decided impression was now made upon his system, at the same time using such local applications as were necessary for throat affections. About the 12th of the month I began to feel my patient improving, and regaining his voice and spirits; and, by the latter part of the month, the throat was well; so he was ordered the "mixed treatment," to be continued many months, and enjoined to commit no excesses in drinking or otherwise, but to maintain the standard of high health. This patient remained well and vigorous for some time, but came to visit me in July, 1869, when I was absent from the city, and, upon my return in August, gave me a call to exhibit a small periosteal node upon left tibia, and excessive surface tenderness of both bones, with, I thought, some sub-periosteal effusion. The iodide potassium, with bark mixture, in fair but increasing doses to saturation, nutritious diet, bathing, etc. In a few weeks he was apparently well, only complaining of occasional nocturnal pains, and slight tenderness upon pressure, enhanced by damp, rainy or windy weather. I attempted to persuade him to winter at the Hot Springs, in Arkansas, as I knew the virtues of those waters unsurpassed in their curative properties upon all blood diseases.

There is one fact to be noted, however, in regard to the failures in many instances among those who visit these celebrated springs, and which is generally laid upon the wrong shoulders. Patients go there, and, having plenty of leisure time, begin to play "draw poker," consume large quantities of bad whisky, neglect the treatment and regimen instituted by the local physicians, and, after a few months' experience, become disgusted and sceptical. They should remember that, at home, especially in Chicago, they could have as much "draw poker," bad whisky, and a greater abundance of all good and bad things, than in any other city of its size in the universe; and it would seem to be an unnecessary and unreasonable waste of time and money to seek further.

Case 3rd presents another phase of this Protean disease. A bubo (*d'emblée*) as the primary accident, followed by secondary and tertiary phenomena. This doctrine was accepted, and held sway for many years among many of the best physicians in France and Germany; but it has gradually lost ground, and few can be found, now-a-days, to uphold it. Such apparent inconsistencies arise, generally, not so much from the still-existing obscurity as to the essence of this disease, but to our lack of skill, ignorance of its history and effects, and want of proper observation.

Moral: In the sere and yellow leaf, seek not Aspasia; *now* the Oceanidæ should be impossible.

